# Reporting standards for guideline-based performance measures

**DOI:** 10.1186/s13012-015-0369-z

**Published:** 2016-01-15

**Authors:** Monika Nothacker, Tim Stokes, Beth Shaw, Patrice Lindsay, Raija Sipilä, Markus Follmann, Ina Kopp, on behalf of the Guidelines International Network (G-I-N) Performance Measures Working Group

**Affiliations:** 1 Arbeitsgemeinschaft der Wissenschaftlichen Medizinischen Fachgesellschaften—Institut für Medizinisches Wissensmanagement (AWMF-IMWI), Philipps-Universität Marburg, Karl-von-Frisch-Str.1, Marburg, 35043 Germany; 2 Department of General Practice and Rural Health, Dunedin School of Medicine, University of Otago, Dunedin, New Zealand; 3 National Institute for Health and Care Excellence (NICE), Manchester, UK; 4 Heart and Stroke Foundation, Ottawa, Canada; 5 The Finnish Medical Society Duodecim, Current Care Guidelines, Helsinki, Finland; 6 German Cancer Society, Berlin, Germany

**Keywords:** Guideline, Guideline adherence, Performance measure, Quality indicator, Reporting standard, Process assessment (health care), Delphi technique

## Abstract

**Background:**

The Guidelines International Network (G-I-N) aims to promote high quality clinical guideline development and implementation. Guideline-based performance measures are a key implementation tool and are widely used internationally for quality improvement, quality assurance, and pay for performance in health care. There is, however, no international consensus on best methods for guideline-based performance measures. In order to address this issue, the G-I-N Performance Measures Working Group aimed to develop a set of consensus-based reporting standards for guideline-based performance measure development and re-evaluation.

**Methods:**

Methodology publications on guideline-based performance measures were identified from a systematic literature review and analyzed. Core criteria for the development and evaluation process of guideline-based performance measures were determined and refined into draft standards with an associated rationale and description of the evidence base. In a two-round Delphi-process, the group members appraised and approved the draft standards. After the first round, the group met to discuss comments and revised the drafts accordingly.

**Results:**

Twenty-one methodology publications were reviewed. The group reached strong consensus on nine reporting standards concerning: (1) selection of clinical guidelines, (2) extraction of clinical guideline recommendations, (3) description of the measure development process, (4) measure appraisal, (5) measure specification, (6) description of the intended use of the measure, (7) measure testing/validating, (8) measure review/re-evaluation, and (9) composition of the measure development panel.

**Conclusions:**

These proposed international reporting standards address core components of guideline-based performance measure development and re-evaluation. They are intended to contribute to international reporting harmonization and improvement of methods for performance measures. Further research is required regarding validity, acceptability, and practicality.

**Electronic supplementary material:**

The online version of this article (doi:10.1186/s13012-015-0369-z) contains supplementary material, which is available to authorized users.

## Background

Clinical practice guidelines aim to improve the quality of patient care [1]. The Institute of Medicine (IOM) defines quality of health care as “the degree to which health services for individuals and populations increase the likelihood of desired health outcomes and are consistent with current professional knowledge” [2], implying a dynamic relation between structure, process, and outcome [3, 4]. The development and use of performance measurement is recommended as one important way to reach improvement [1]. During the last decade, an exponential increase in the number of performance measures (PM) used in health care has occurred, leading to concerns about their trustworthiness and usefulness [5]. In 2012, more than 2000 PM were published via the National Quality Measures Clearinghouse, tenfold more than in 2003 [5].

Existing guidance on PM development or appraisal highlights that clinical guidelines are an important source for quality measures [6–11]. From a guideline implementation perspective, PM are important tools as they enable standardized measurement of the extent of implementation and the effectiveness of specific recommendations contained within guidelines [12, 13]. We lack, however, any international consensus as to the most appropriate methods for guideline-based PM [14, 15].

The Guidelines International Network (G-I-N) (http://www.g-i-n.net/) aims to lead, strengthen, and support collaboration in guideline development, adaptation, and implementation [16]. In 2012, a working group on PM was set up to bring guideline and PM developers together. The G-I-N PM working group (PMWG) consisted of 30 members of organizations producing or evaluating guideline-based PM from 13 countries in 5 continents. Approximately two thirds were mainly guideline and one third mainly PM experts. The group considered that the promotion of a high-quality guideline-based PM methodology required a set of “best practice” reporting standards. This paper presents the consensus of our group on reporting standards and describes the methods used to develop them.

## Methods

(The methods are described in detail in the study protocol; see Additional file 1).

### Literature review and identification of core criteria

In order to identify candidate core criteria, we searched for relevant publications on the development of guideline based PMs. We identified one systematic review, judged to be of good quality using the AMSTAR checklist [17], with 48 publications on guideline-based PM [14] (appraisal results in Additional file 2). We considered all publications included in this review and agreed 16 as relevant for this work, described in the original review as “methods papers.” As the review search ended on 22/04/2010, we performed specific update searches in Medline via PubMed from 23/04/2010 to 08/12/2013 using the same search strategy (see Table 1) [14]. We included methodology publications for guideline-based PM in English, French, and German. Additionally, we reviewed the currency of web-based guidance manuals for PM development in November 2013 and included hand-searched publications, suggested by two members of the PMWG.

**Table 1:** Search strategy for guideline-based performance measures used by Kötter et al. 2012^14^

Quality indicators	Clinical guidelines	Development
1. quality indicator$.tw	12. guideline$.tw	31. develop$.tw
2. quality criterion$.tw	13. practice guideline/	32. and\11,30,31
3. quality measure$.tw	14. practice guideline$.tw	
4. performance indicator$.tw	15. clinical practice guideline$.tw	
5. performance measure$.tw	16. recommendation$.tw	
6. outcome measure$.tw	17. guidance$.tw	
7. outcome indicator$.tw	18. directive$.tw	
8. audit.tw	19. health service$ research.tw	
9. outcome assessment.tw	20. evidence based medicine.tw	
10. process assessment.tw	21. quality assessment.tw	
11. or\1-10	22. quality assurance.tw	
	23. consensus technique.tw	
	24. delphi technique.tw	
	25. RAND.tw	
	26. UCLA.tw	
	27. RAM.tw	
	28. RAND appropriateness method.tw	
	29. consensus development/	
	30. or\12–29	

The steps of guideline-based PM development set out in the review were used to identify potential core reporting criteria [14]. The criteria were critically reviewed, refined, and complemented as well as supplemented with rationales by MN, TS, and BS based on the results of the review and the update search. The draft set of criteria and attendant rationales was initially reviewed by four international experts from AHRQ (Agency for HealthCare Research and Quality), AWMF (Association of the Scientific Medical Societies in Germany), SIGN (Scottish Intercollegiate Guidelines Network), and the Heart and Stroke Foundation of Canada.

### Delphi process

In the next step, we invited the members of the PMWG to a Delphi process, a written formal technique for reaching a consensus in a group of experts. It is characterized by the use of structured questionnaires and formal feedback of the group result, summarizing the answers and comments to each question. It usually requires at least two rounds allowing individual participants to reconsider their views in the light of the group decision and arguments [18]. We used an online survey. In the first round, participants were asked to rate each criterion and rationale on a four-point Likert-type scale “I agree, I rather agree, I rather disagree, I disagree” and in addition, to rate the importance of each criterion on a respective Likert Scale (critically important, important, minor important, not important). To reach consensus, the overall agreement for a criterion implied at least 75 % of participants to have scored “agree” or “rather agree.” Group members could also give comments and propose modifications as well as additional criteria which they considered being essential. Results were discussed in a PMWG meeting. In the second round, participants were again asked to rate agreement and importance.

## Results

**Fig. 1:**
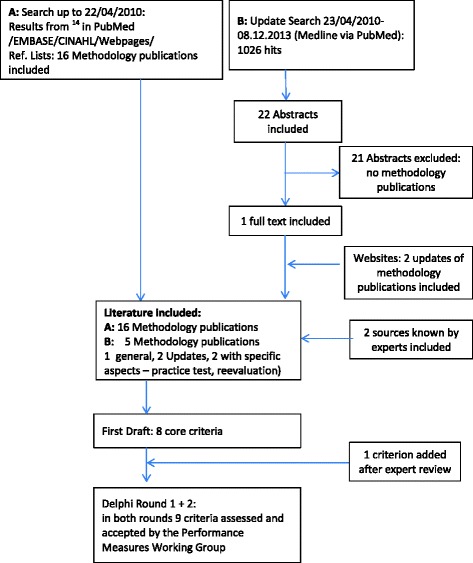
Literature review and consensus process for reporting criteria on guideline-based performance measures

In total, 21 methodology publications were included. Sixteen methodology publications were identified by the authors of the systematic review [14]; these were agreed as being relevant to this work [19–34]. The update search yielded three additional methodology publications [35–37]. Two publications reporting approaches to guideline-based PM from Netherlands and Germany were contributed by PMWG members [8, 38] (see Fig. 1).

One criterion (topic selection) included in the systematic review [14] was excluded for our purposes as not being core to guideline-based PM reporting. A further two criteria were added (3 and 4), based on our own experience and knowledge and accepted best practice in PM development. Another criterion (specification of PM) was added as the result of the experts’ comments. Thus, nine criteria were proposed.

Of the 30 experts invited to participate in round 1, 90 % responded and 70 % (21/30) completed the online Delphi questionnaire. The 27 respondents of round 1 were invited to take part in round 2. Of those, 44 % (12/27) completed the rating of the criteria and approved the rationales (see Additional files 3 and 4). Editorial changes during the publication process were approved by the group.

**Fig. 2:**
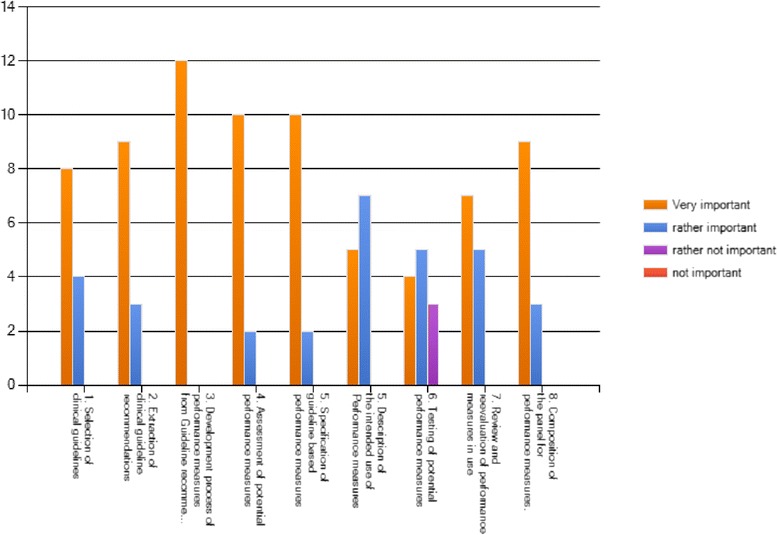
Assessment of importance of the reporting criteria

In Delphi round 1, more than 75 % overall agreement was reached for all criteria (between 77 and 100 % per criterion scored with “agree” or “rather agree”). No additional criteria were proposed. The main outcome of the discussion in between the Delphi rounds was the need for consistent wording expressing the goal of developing reporting standards as opposed to developing a quality appraisal tool. Revision of the criteria was made accordingly. In Delphi round 2, the overall agreement for every revised criterion and rationale was at least 90 % (see Table 2). The importance awarded for the criteria varied, but consensus was reached for the importance with more than 75 % of the participants rating all criteria as “very important” or “rather important” (see Fig. 2). Based on these results and subsequent editing to align these standards with wording in other reporting standards [67], the G-I-N PMWG presents the following reporting standards for guideline-based performance measures (see also Table 2).

**Table 2:** Criteria for development of guideline-based performance measures, supporting methodology publications and strength of consensus at Delphi round 2

Criterion	Supporting methodology publications as identified by systematic search	Strength of consensus
1. Guideline selection 1a. State the currency of the guideline(s) used for guideline-based performance measure development and state if it/they meet the criteria set out by the Guidelines International Network (G-I-N). Describe the guideline quality using a validated guideline appraisal tool, such as AGREE II. b. Indicate additional sources, if used and the rationale for their use.	AQUA 2013 [35] Campbell 2002 [22] 1b: ACCF/AHA 2005 [28]/2010 [37] ÄZQ 2009 [24] Graham 2009 [27] Hutchinson 2003 [30]	Overall agreement 100 % Agree 67 % Rather agree 33 %
2. Selection of guideline recommendations State the strength of evidence and/or the grade of recommendation qualifying the guideline recommendations to be used for guideline-based performance measures.	AHCPR 1995 [20] AHCPR 1995 [19] ACC/AHA 2005 [28]/2010 [37] ÄZQ 2009 [24] AQUA 2010 [23] /2013 [35] Baker 1999 [21] Bergman 1999 [25] Califf 2002 [26] Campbell 2002 [22] Duffy 2005 [32] Graham 2009 [27] Hutchinson 2003 [30] De Koning 2006 [8] Wollersheim 2003 [29]	Overall agreement 100 % Agree 75 % Rather agree 25 %
3. Selection process of performance measures from guideline recommendations Describe clearly and in detail the methods used to develop the performance measures from the supporting clinical guideline recommendations.	ACC/AHA 2005 [28] ACCF/AHA 2010 [37] ÄZQ 2009 [24] AQUA 2010 [23]/2013 [35] Califf 2002 [26] Campbell 2002 [22] Campbell 2011 [36] Duffy 2005 [32] Hutchinson 2003 [30] De Koning 2006 [8] LaClair 2001 [34]	Overall agreement 100 % Agree 92 % Rather agree 8 %
4. Core attributes of performance measures State, if the following attributes within the development process of guideline-based performance measures were considered: • Relevance (as a minimum: potential for improvement/clinical relevance) • Scientific Soundness (as a minimum: the evidence supporting the measure) • Feasibility (as a minimum: clarity of definition and measurability)	ACC/AHA 2005 [28] ACCF/AHA 2010 [37] ÄZQ 2009 [24] Baker 1995 [21] Califf 2002 [26] Graham 2009 [27] Campbell 2002 [22] Campbell 2011 [36] Duffy 2005 [32] Golden 2008 [33] Wollersheim 2003 [29]	Overall agreement 100 % Agree 92 % Rather agree 8 %
5. Specification of performance measures State that numerator and denominator of the guideline-based Performance Measure is specified unambiguously and in detail.	ACCF/AHA 2005 [28] Advani 2003 [31] ÄZQ 2009 [24] AQUA 2010 [23] /2013 [35] Campbell 2002 [22] Campbell 2011 [36] Golden 2008 [33] LeClair 2001 [34]	Overall agreement 100 % Agree 100 %
6. Intended use of performance measures State if there is a clear description of the intended use of the performance measure (quality improvement, quality assurance with or without accountability purposes, pay for performance) and at what level in the health system it is used (local, regional, national).	ACCF/AHA 2010 [37] ÄZQ 2009 [24] AQUA 2013 [35 Campbell 2002 [22] Campbell 2011 [36]	Overall agreement 100 % Agree 75 % Rather agree 25 %
7. Practice test of performance measures If a practice test (piloting) is carried out prior using the guideline-based performance measure, provide a full description of the process. If no practice test is done, provide the rationale for this. Provide information about any other validation process in use.	AHCPR 1995 [20] AHCPR 1995 [19] AQUA 2013 [35] Campbell 2011 [36] ACCF/AHA 2005 [28] Golden 2008 [33] Wollersheim 2003 [29]	Overall agreement 100 % Agree 75 % Rather agree 25 %
8. Review and reevaluation of performance measures Report the currency of the performance measures in use. State if there are criteria for deciding to change or stop using performance measures.	Graham 2009 [27] ACCF/AHA 2010 [37] Duffy 2005 [32] Follmann 2012 [38]	Overall agreement 92 % Agree 75 % Rather agree 17 % Rather disagree 8 %
9. Composition of the panel deciding on guideline-based performance measures Describe clearly the composition of the panel deciding on guideline-based performance measures with information on participation of multidisciplinary experts, stakeholders in the field, experts in quality measurement, and patient representatives.	ACCF/AHA 2005 [28] ÄZQ 2009 [24] AQUA 2010 [23] /2013 [35] Campbell 2002 [22] Campbell 2011 [36] Duffy 2005 [32] Hutchinson 2003 [30] Wollersheim 2003 [29]	Overall agreement 100 % Agree 92 % Rather agree 8 %

### 1. Guideline selection

a. State the currency of the guideline(s) used for performance measure development and state if it/they meet the criteria set out by the Guidelines International Network (G-I-N). Describe the guideline quality using a validated guideline appraisal tool, such as AGREE II.

b. Indicate additional (evidence) sources, if used and the rationale for their use.

#### Rationale

To provide a high quality, trustworthy base for PM, the source clinical guideline should meet the criteria published by G-I-N [39] (i.e., use of a multidisciplinary development group, description of the decision-making process and its methods, declaration and management of conflicts of interest, systematic evidence reviews, and clear wording of recommendations). In order to objectively evaluate the guideline quality and allow comparison against other source guidelines, a validated appraisal instrument (preferably the Appraisal of Guidelines for Research and Evaluation (AGREE) instrument II [13]) should be used; in the case of guideline adaptation an internationally accepted instrument, preferably ADAPTE [40] is recommended. A crucial fact to consider is the currency of the guideline to ensure consistency with the actual evidence base of guideline recommendations under consideration for the development of performance measures. In case of doubt, an update search of the literature is recommended [41].

Existing PM should also been taken into account as evidence sources when developing “new” indicators. There are national and regional databases and other sources for existing PM. If additional evidence sources are used, the rationale for their use should be clearly stated.

A full description and quality appraisal of the underlying guideline resource and, if applicable, the citation of other evidence resources are not currently standard practice in publications of guideline-based PM which are not developed simultaneously with a guideline. In addition, only about half of the publications of the systematic review provide a methodological quality appraisal of the guidelines used [14].

### 2. Selection of guideline recommendations

State the quality of evidence and/or the strength of recommendation qualifying the guideline recommendations to be used for performance measure development.

#### Rationale

PM are based on specific guideline recommendations or objectives for care. Recommendations predominantly address health care processes (e.g., diagnostic and therapeutic interventions) which ideally are under the control of health care professionals. Structures and outcomes of care are more determined by influencing systemic factors and regional or national conditions [42].

Internationally, there is debate about the type of recommendations which are adequate sources for PM. Recommendations with a strong grade of recommendation (“we recommend,” “do,” “should be done” or “we don’t recommend,” “don’t do,” “should not be done”) are meant to be useful and valid for the majority of patients whereas weak recommendations describe options for individual situations. Recommendations with a weak grade of recommendation (“we suggest,” “probably do,” “can be considered,” “might be done”) are therefore not considered suitable for PM intended to serve for external quality assurance, in particular accountability purposes (e.g., pay for performance). The strength of a recommendation does not only express the level of the underlying evidence but also other considerations like the level of confidence that implementing this recommendation will do more good than harm or will avoid harm in case of negative recommendations [43, 44]. Strong recommendations should ideally be based on high quality evidence, but if evidence is of poor quality or absent, which is the reality for various aspects in health care that are most important for patients, a strong expert consensus might also be considered as a source for a guideline-based PM, given this was reached by a multidisciplinary panel using formal techniques to reduce the risk of bias [9, 22, 24]. However, the inherent uncertainty about the effects of such PM requires their piloting before broader implementation (see criterion 7).

Levels of evidence and/or grade of recommendations of the underlying guideline recommendation are provided in about two third of respective publications [14].

### 3. Selection process of performance measures from guideline recommendations

Describe the consensus methods used to select the performance measures from the supporting clinical guideline recommendations in detail.

#### Rationale

The process of selection implies specification of candidate PM (see criterion 5), critical appraisal according to the specific criteria or core attributes (see criterion 4), and final decision-making by an expert panel (see criterion 9) in a consensus process. To minimize bias, it is recommended to use formal consensus methods [8, 22–24, 26, 28, 30, 32, 34–37].

Appraisal of guideline-based PM should be done in several steps. Some attributes can be appraised directly, before implementing the measure (see criterion 4); others can only be appraised when there are already data of the respective measure available (e.g., reliability, validity).

Reporting the consensus methods used for selection and how they were conducted helps to assess the quality of the selection process. To our knowledge, there are presently no studies comparing different selection methods. The majority of publications identified stated the use of formal consensus methods to develop PM. Nearly half of the studies available cite the Research and Development Corporation (RAND) Appropriateness Method (RAM) developed by the RAND and the University of California, Los Angeles (UCLA) or a modified RAM using the consensus method with varying appraisal criteria and cutoff levels for agreement. Also mentioned is a RAND-modified Delphi method or a Delphi process [14].

### 4. Core attributes of performance measures

State the consideration of the following attributes during the performance measure development process:

- Relevance (as a minimum: potential for improvement/clinical relevance)
- Scientific Soundness (as a minimum: the evidence supporting the measure)
- Feasibility (as a minimum: clarity of definition and measurability)

#### Rationale

Frequently reported appraisal criteria in the methodology publications include usefulness of PM for improving patient outcomes, relevance, and feasibility of monitoring [20, 22, 24, 26, 28, 32, 35, 37]. A comparison of internationally used PM appraisal criteria found the criteria “relevance” or “needs assessment”/prioritization as well as clarity of definitions, feasibility, reliability, and validity in all four national approaches analyzed [35] (the Quality and Outcomes Framework (QOF) led by the UK National Institute for Health and Care Excellence (NICE), the German appraisal instrument QUALIFY by the Institute for Quality and Patient Safety, the approach of the approach of the American-based National Quality Forum and the criteria used by the Royal Australian College of General Practitioners). Acceptability was stated as criterion in two of them [36]. A further national indicator assessment tool—the Dutch “Appraisal of indicators through research and evaluation” (AIRE) instrument, does not name “relevance” but covers feasibility as well as reliability and validity [8]. The US National Quality Measures Clearinghouse suggests categorizing the attributes of PM in three domains: (1) importance, (2) scientific soundness, and (3) feasibility [45]. Where feasibility and most scientific soundness criteria (e.g., reliability and validity) can only be verified through a practice test, the importance or relevance of a measure, its clarity of definition (defining numerator and denominator unambiguously), and the evidence supporting the measure might be assessed by experts without specific PM data [24].

Given different possible perspectives, developers should explain which aspects they summarize under “relevance” or “importance” of a measure” (e.g., potential benefit for improvement of patient outcomes, relevance for the specific health system, cost saving, etc.). Further appraisal and selection after having done a practice test (e.g., does it measure what it should measure—reliability and validity, as well as feasibility in current systems) is reasonable. If a recommendation is potentially measurable, the lack of measurement feasibility in current health information systems should not be used as the sole criterion for determining a measure to be invalid as improvements to existing data can result in a measure becoming feasible.

### 5. Specification of performance measures

Specify the performance measure numerator and denominator unambiguously and in detail.

#### Rationale

To be measurable, a guideline recommendation has to be transformed into a rate or proportional based measure consisting of numerator and denominator. Only in a few cases, PM consist of so-called sentinel events which should be presented as counts, rather than proportions—for example, maternal deaths [22]. Inclusion criteria for the denominator have to specify the patient characteristics (e.g., age, gender, stage, or severity of disease, having had a certain treatment already) in a way that patients to be included can clearly be identified and data—ideally in an electronic system, can be collected. The same specifications have to be made for the numerator: the diagnostic test or therapeutic intervention has to be described and specified clearly without ambiguity. In addition, the specific date or period of measurement has to be noted. For all specifications, a list of data fields has to be set up to ensure an adequate and consistent documentation. This list should be provided as accompanying information for each PM. It is also crucial to define possible exceptions for both numerator and denominator (e.g., age, contraindications, technical obstacles, patient wish). If possible, PM should be integrated into existing coding and data systems, as parsimonious PM and data use is an important goal.

#### Example of a rate based measure

Recommendation: For patients with R0 resected stage III colon cancer, adjuvant chemotherapy is indicated. (Level of Evidence 1a, strong consensus)

Numerator: Number of patients who have undergone adjuvant chemotherapy.

Denominator: All patients with colon cancer Union internationale contre le cancer (UICC) stage III who have had an R0-resection of the primary tumor.

Further specification: substances, minimal number of chemotherapy cycles required, timeframe.

Exceptions: Patient refusal, contraindications to be specified.

### 6. Intended use of performance measures

Provide a clear description of the intended use of the performance measure (e.g., quality improvement, quality assurance with or without accountability purposes as pay for performance, public reporting) and at what level in the health system it is used (local, regional, national).

#### Rationale

The intended use of guideline-based PM should be stated as PM are to be used at different levels within health systems:

- Quality improvement (QI)—for internal quality improvement purposes such as voluntary initiatives of health professionals (e.g., local clinical audit, peer review)
- Accountability—containing different aspects, for example:
  Certification: for quality assurance at a regional or national level, such as being used as a compulsory instrument for quality assurance in hospitals and/or a defined ambulatory care setting or for various aspects of the in- and/or out-patient sector in general [46]. Important aspects of the use of such PM are benchmarking and public reporting [47].
  Pay for performance, where payment is attached to defined levels of achievement of the measure with the intention of improving the quality of care [48, 49].

In addition, quality measures may be used in research to develop or produce new knowledge about the health care system.

The different uses of PM influence the indicator development methods that have to be applied. It seems useful to distinguish PM into those to be used for QI and those used for accountability purposes [50, 51]. A key difference between measures developed for quality improvement and those developed for public reporting or accountability purposes is that the requirements for validity and reliability are more complex for the latter as they include different denominators (population based vs local patient based rates) [11]. Accountability measures require that each provider collects data in exactly the same way through standardized and detailed specifications. This ensures that one is confident that a predefined measure of performance has been achieved and/or that comparisons of performance between providers are fair. Beyond that, risk adjustment is essential. It is used to compensate for factors like age and health burden-related differences in the patient mix at various sites in order to make the results from different sites fairly comparable [9, 22]. Thus, it is important that the rigor of PM development (as assessed by 4 and 5) reflects their intended use in the health system [50, 52].

### 7. Practice test of performance measures prior to their broader implementation and routine use

Provide a full description of the practice test (piloting) prior to using the guideline-based performance measure. If no practice test is done, provide the reason for this. Provide information about any other validation process in use.

#### Rationale

All measures but especially indicators that are used for regional or national reporting purposes or pay for performance are at risk of having unintended consequences [53, 54]. A practice test offers the opportunity to identify such unintended consequences early. Practice tests are recommended in the early methods concepts for guideline-based PM by Agency for Health Care Policy and Research (AHCPR) [11, 19, 20, 55], and testing is described in the AHRQ-report of Performance Measure Development 2011 [11, 19, 20, 55]. There is no standard definition of what a practice test has to contain. A comprehensive piloting method was introduced to the nationwide UK Quality and Outcomes Framework (QOF) after experience of unintended consequences without such a procedure [36, 56]. Another nationwide PM program includes a three step piloting and practice test [35]. Practice tests (piloting) of guideline-based PM are only described in a minority of published projects [14, 57]. The methods chosen for the practice test should be in accordance with the intended use of the measures (see 4). It should be done before the final decision of use is made. It is recommended that testing should be done in a representative “real world” setting. The process for final decision-making to use or not to use the PM should be transparent. PM require monitoring when implemented in order to evaluate their longer term appropriateness.

### 8. Review and reevaluation of performance measures

State the currency of the performance measures in use. State the criteria for deciding to change or stop using performance measures.

#### Rationale

There can be several reasons to stop a measurement, for example, if measures show unintended consequences or lack of reliability [35]. Other reasons to reconsider the use of an indicator are given if the evidence base changed or if the defined performance “benchmarks” are reached and are stable over a defined period of time [37, 38, 58]. In order to promote transparency, “stop” criteria should be stated explicitly. If data are available, ideally, the underlying guideline and the PM should be updated simultaneously [38].

### 9. Composition of the panel deciding on guideline-based performance measures

Describe clearly the composition of the panel deciding on the performance measures with information on participation of multidisciplinary experts, stakeholders in the field, experts in quality measurement, and patient representatives.

#### Rationale

Similar principles apply to the composition of guideline-based PM groups as to clinical practice guidelines, including the consideration of conflicts of interest, but there is very little evidence for this rationale. According to the “good practice,” the group should be multidisciplinary and include content experts in the field—usually health care professionals involved in the relevant clinical guideline development including stakeholders who are being measured and, if applicable and possible stakeholders who will use this data to inform decisions, patient representatives and experts in quality measurement (representing the organizations which measure) [13]. This is also a criterion of the Dutch AIRE instrument [59]. Only a few studies named the individual members of the panels [14] whereas criteria for their selection (e.g., clinical or methodological expertise, membership in a specialist society) were reported in most of the studies. Patient participation during the development process was only reported in few studies, in all of these patients participated directly in the panels. No study reported on patient participation during guideline selection and the extraction of recommendations [14]. The best method of involving patients remains a subject for further research [60].

## Discussion

We have developed a reporting standard for guideline-based PM with nine criteria, using formal written consensus methods (two Delphi rounds). This is the first work to our knowledge presenting a consensus on transparent reporting of methodological requirements of guideline-based PM by an international group. Applying these criteria aims to make guideline-based PM more comprehensible and valuable. The fact that all criteria were confirmed after the first round indicates their international meaningfulness for the participating guideline and PM experts. There was a strong view on these criteria to be used as standard reporting criteria. The group judged the available evidence not yet sufficient for the development of an appraisal tool.

The methodology publications identified were heterogeneous concerning criteria for guideline selection, selection of guideline recommendations, panel selection, consensus methods, appraisal criteria, and proposals for practice tests. No paper included and specified all nine criteria presented here. In light of missing comparative studies, the structured consensus process was most important to ensure the best possible expert-based PM reporting criteria. It is notable that one of the steps in PM development identified in the systematic review [14]—“topic selection”—was not included in the reporting standard. The rationale for this was that while it was judged essential to assess potential PM based on guideline recommendations according to their relevance for health care (see criterion 4), topic selection should occur before guideline-based PM development starts. In general, prevalence, burden of disease, and potential of improvement are well-accepted criteria for topic selection of clinical guidelines [61, 62].

We identify the following limitations of this work: First, the present criteria do not address any qualitative aspects of patient care to be reported. It is recognized that different patient groups do have different needs and the best quality of care may be different for the same condition, requiring differentiated patient-oriented measures (for example, for patients with multiple morbidities) [63]. Second, we also do not address outcome measurement development and therefore patient reported outcome measures, including quality of life measures are not mentioned [64]. Guideline-based patient reported outcomes or quality of life measures are up to now almost completely missing in guideline-based PM [65]. Third, there was no consideration of cost-effectiveness to be a criterion for the use of a guideline-based PM [66]. For these aspects, further research is required.

Furthermore, the consensus process was done with a small group of experts with a low participation rate in the second round. However, there was a high acceptance already in the first round and the final draft was approved by the whole group.

Finally, not all criteria were judged to be of similar importance (see Fig. 2).

It is planned that the reporting standards will be tested in different countries and settings in order to evaluate their validity, acceptability, and practicality.

## Conclusion

These reporting standards provide international consensus on the best practice criteria for reporting guideline-based PM development and re-evaluation. Better reporting of methods used by PM developers should improve both the quality and consistency of guidelines-based PM. The PMWG encourages research on the validity, acceptability, and practicality of the respective criteria.

## Additional files

Additional file 1: **Study protocol (2013).** (PDF 427 kb)

Additional file 2: **AMSTAR appraisal of the systematic review Kötter et al., 2012.** (PDF 13 kb)

Additional file 3: **G-I-N survey round I, copy of survey monkeys results and comments.** (PDF 366 kb)

Additional file 4: **G-I-N survey round 2; copy of results.** (PDF 266 kb)

## Abbreviations

AGREE: Appraisal of Guidelines for Research and Evaluation
AHCPR: Agency for Health Care Policy and Research
AHRQ: Agency for Health Care Research and Quality
AIRE: Appraisal of indicators through research and evaluation
AWMF: Association of the Scientific Medical Societies in Germany
G-I-N: Guidelines International Network
IOM: Institute of Medicine
NICE: National Institute for Health and Care Excellence
PM: performance measure
PMWG: Performance Measures Working Group
RAM: RAND Appropriateness method
RAND: Research and Development Corporation
QOF: Quality and Outcomes Framework
SIGN: Scottish Intercollegiate Guidelines Network
UCLA: University of California, Los Angeles
UICC: Union internationale contre le cancer (Organization that provides cancer classification systems)
