# Supplementary material for: Reporting standards for guideline-based performance measures
Source: Implement Sci. 2016 Jan 15;11:6. doi: 10.1186/s13012-015-0369-z (PMC4714427; doi:10.1186/s13012-015-0369-z)
Supplement: Supplementary file 1 — Study protocol (2013). (PDF 427 kb) [file 13012_2015_369_MOESM1_ESM.pdf]

Guideline Based Performance Measures: Towards G-I-N Standards

1. Background

1.1 Guidelines and Performance Measures

The principal aim of clinical guidelines is to improve the quality of patient care. According to the current definition of the Institute of Medicine (IOM), clinical practice guidelines are "statements that include recommendations intended to optimize patient care that are informed by a systematic review of evidence and an assessment of the benefits and harms of alternative care options"[1]. The IOM defines quality of health care as "the degree to which health services for individuals and populations increase the likelihood of desired health outcomes and are consistent with current professional knowledge."[2] This definition implies an influenceable relation between structure, process and outcome of health care[34]. To answer the question if patient care is optimized by guideline recommendations, performance measures are essential for evaluating compliance with guideline recommendations as well as for analyzing the correlation between the performance of structures and processes and the desired health outcome. Results of performance measures can also be used for analysis of acceptance, relevance and practicability of guideline recommendations, thus contributing to a "plan-do-check-act" quality improvement circle for the guideline itself[5] when feedback is given to guideline authors.

1.1.1 Scope of guideline based Performance Measures

Guideline recommendations are predominantly addressing processes of health care (a.o. diagnostic and therapeutic interventions) in contrast to structures or outcomes. Processes can be best directly influenced by health care professionals. Structures and especially outcomes may underlie more multifaceted influencing factors [6]. The assumption of an influenceable relationship between quality of structure, process and outcome can therefore not be considered a simple linear relationship[7].

[1] Institute of Medicine. Clinical Practice Guidelines We Can Trust. Washington,DC: National Academies Pr; 2011.

[2] http://www.iom.edu/Global/News%20Announcements/Crossing-the-Quality-Chasm-The-IOM-Health-Care-Quality-Initiative.aspx [10.12.2012]

[3] Donabedian A (1966) Evaluating the quality of medical care. Milbank Memorial Fund Quarterly: Health Soc44(3; pt. 2):166–203

[4] Kopp IB (2011) [From clinical practice guidelines towards quality assurance]. Bundesgesundheitsblatt Gesundheitsforschung Gesundheitsschutz. 2011 Feb; 54(2):160-5. German.

[5] Kopp IB (2011) [From clinical practice guidelines towards quality assurance]. Bundesgesundheitsblatt Gesundheitsforschung Gesundheitsschutz. 2011 Feb; 54(2):160-5. German.

[6] Nothacker MJ, Langer T, Weinbrenner S. [Quality indicators for National Disease Management Guidelines using the example of the National Disease Management Guideline for "Chronic Heart Failure"]. Z Evid Fortbild Qual Gesundhwes. 2011;105(1):27-37. doi: 10.1016/j.zefq.2010.07.003. German.

[7] Lit. Will be added

1.1.1 Use of Performance Measures

The IOM recommended in 2011 that "incorporation of performance and outcome measurements for improvement and accountability" was an important way to deliver the six identified aims for health care improvement (Safety, effectiveness, patient centeredness, timeliness, efficiency and equity)[8]. A variety of initiatives use performance measurement on different levels. First, performance measures are used for quality improvement purposes such as voluntary initiatives of health professionals for internal quality management[910].

Second, they can be used for quality assurance without or with accountability purposes at regional or national level: such as being used as a compulsory instrument for quality assurance in hospitals and/or a defined ambulant care setting or for various aspects of the in- and/or out-patient sector in general[11]. An important aspect of the use of such performance measures is benchmarking[12] and public reporting[13]. Third, the performance measures may be used as part of pay for performance (where is payment attached to defined levels of achievement of the measure with the intention of improving the quality of care ([1415]). These different uses of performance measures influence the indicator development methods that are applied.

1.2 The Guidelines International Network

The mission of the Guidelines International Network (G-I-N) is "to lead, strengthen and support collaboration and work within the guideline development, adaptation and implementation community" [16]. One of the aims of G-I-N is to promote best practice and to establish high quality standards of guideline development as well as guideline implementation. To meet this aims, a possible action outlined in the current G-I-N strategic plan (G-I-N strategic plan 2012-2013[17] is setting standards. Whereas the G-I-N Board published 2012 a set of key components for clinical practice guidelines focusing on the guideline development process[18], for the development of guideline based performance measures so far no international standard has been formulated.

---

[8] Institute of Medicine. Crossing the Quality Chasm: A New Health System for the 21st Century, http://www.nap.edu/catalog/10027.html [22.01.2013] „Health Care should be safe, effective, patient centered, timely, efficient and equitable"

[9] Andres E, Beyer M, Schorsch B, Szecsenyi J, Dryden WA, Kreuz I, Kunze MT, Tischer KW, Gerlach FM.
[Quality circles in German ambulatory care: results of a continuous documentation in the regions of Bremen, Saxony-Anhalt, Schleswig-Holstein and Westphalia-Lippe 1995-2007].Z Evid Fortbild Qual Gesundhwes. 2010;104(1):51-8. German.

[10]

[11] Lit. will be added

[12] Brucker SY, Wallwiener M, Kreienberg R, Jonat W, Beckmann MW, Bamberg M, Wallwiener D, Souchon R.Optimizing the quality of breast cancer care at certified german breast centers: a benchmarking analysis for 2003-2009 with a particular focus on the interdisciplinary specialty of radiation oncology.Strahlenther Onkol. 2011 Feb;187(2):89-99. doi: 10.1007/s00066-010-2202-6. Epub 2011 Jan 21.

[13] Lit. will be added

[14] Doran T, Kontopantelis E, Valderas JM, Campbell S, Roland M, Salisbury C, Reeves D Effect of financial incentives on incentivised and non-incentivised clinical activities: longitudinal analysis of data from the UK Quality and Outcomes Framework. BMJ. 2011 Jun 28;342:d3590. doi: 10.1136/bmj.d3590.

[15] Kruse GB, Polsky D, Stuart EA, Werner RM. The impact of hospital pay-for-performance on hospital and Medicare costs., Health Serv Res. 2012 Dec;47(6):2118-36. doi: 10.1111/1475-6773.12003.

16 Institute of Medicine. Crossing the Quality Chasm: A New Health System for the 21st Century, http://www.nap.edu/catalog/10027.html [22.01.2013]

17 http://www.g-i-n.net/document-store/g-i-n-strategy-2010-10-13.pdf [10.12.2012]

18 Qaseem A, Forland F, Macbeth F, Ollenschläger G, Phillips S, van der Wees P; Board of Trustees of the Guidelines International Network. Guidelines International Network: toward international standards for clinical practice guidelines.

1.3 Towards a methodological standard for guideline based performance measures: the G-I-N Performance Measures Working Group

There is increasing emphasis on the need for performance measures to be based on quality assured or "trustworthy" clinical guideline recommendations[19]. Recommendations of high quality guidelines are thus an important and timesaving source for performance measures. Performance measure developers and guideline producers can establish partnerships for promoting evidence based health care, meeting another action named in the current G-I-N strategic plan. Such a partnership can promote the use of: a) valid rigorously developed clinical guideline recommendations and b) methodologically sound criteria for measure development, especially concerning relevance, content validity, sensitivity for change and practicality. To date, there is no methodological "gold standard" how to develop valid guideline based performance measures[20]. Even within the G-I-N community, a variety of methods are in use. In a recent survey more than half of the participants stated that the development methods used could be more rigorous[21]. Currently there are various criteria used for the steps of developing performance methods from guidelines.

Thus, there is a clear need for G-I-N to define "best practice" standards for the development of performance measures developed from clinical guideline recommendations which can be used internationally.

While setting up comparative studies is complex and time consuming, a pragmatic solution for setting standards is to develop a set of standards using formal consensus methods with participants being G-I-N members with relevant content expertise.

The following study plan describes the proposed methods for developing consensus standards for the process of developing performance measures from clinical guideline recommendations.

1.4 Using the DELPHI method for formal consensus procedures

Decision-making in groups is accepted as having many advantages including 'safety in numbers', a perception of authority, and rationality (where decisions are improved by reasoned argument in which assumptions are challenged). However, disadvantages of group decision making can be domination of the discussion by particular individuals, or pressures to agree with a majority or powerful person's viewpoint. Formal methods have been developed with the aim of overcoming some of these problems.[22]

The DEPLHI method is one of many formal consensus methods, and is characterized by the use of mailed questionnaires, an elicitation of private decisions, formal feedback of group choices, no face-to-face feedback, structured interaction, and an explicit aggregation method.

2. Aim

Ann Intern Med. 2012 Apr 3;156(7):525-31. doi: 10.1059/0003-4819-156-7-201204030-00009.

[19] Institute of Medicine. Clinical Practice Guidelines We Can Trust. P. 201, Washington,DC: National Academies Pr; 2011.

20 Kötter T, Blozik E, Scherer M. Methods for the guideline-based development of quality indicators--a systematic review. Implement Sci. 2012 Mar 21;7:21. doi: 10.1186/1748-5908-7-21.

21 Blozik E, Nothacker M, Bunk T, Szecsenyi J, Ollenschläger G, Scherer M. Simultaneous development of guidelines and quality indicators -- how do guideline groups act? A worldwide survey.Int J Health Care Qual Assur. 2012;25(8):712-29.

The aim is to develop and agree on a set of core methodological standards for guideline-based performance measures with an associated rationale.

3. Methods

1. Current literature/methods will be taken from the systematic review of Kötter et al, 2012 and a specific update search. Based on approaches in use by already existing programs/ initiatives, core steps for the development process of guideline based performance measures will be identified.
2. A first draft of “best practice” standards will be compiled covering each core step. These will be developed by MN, TS and BS.
3. Each criterion of the draft standard will be justified by a - if available- literature based rationale or by an expert based consensus statement.
4. The methods and the first draft of the standards will be discussed with a small round of experts representing leading organizations for guideline based PM.
5. A formalized DELPHI consensus process[23][24] will be used for agreement respective modifications of the suggested standards within the G-I-N performances measures working group. The criteria for each standard will be assessed anonymously by the group members (agree, critical important; agree, important; agree, minor important; disagree; possibility to agree with suggesting modifications) within a 1st DELPHI Round. The rationale given for the criteria of each step can also be commented and complemented (possible form see appendix 5.1).
6. As the G-I-N group members are heterogeneous concerning their experience with guideline based performance measures as well as their methodological knowledge (of both, guideline methodology and methodology for performance measures), personal experience/knowledge will be asked for in an accompanying survey. If the ratings for the criteria are very divergent there will be an exploration of correlation to personal background and experience.
7. After the 1st DELPHI round, a synopsis of comments and modifications done by the group members will be send to the group together with the revised draft.
8. There will be a discussion by phone or a meeting if there are heterogeneous votes or open discussion points.
9. In a second DELPHI round, the group members will be asked for consent (agree/disagree). Acceptance: at least 75% agree/rather agree.
10. If necessary, in a third DELPHI round, the re-revised standards will be finally assessed (agree/disagree).

4. Results

The results will be presented as recommendations of core components with explanatory statements for the rationale of consideration. The aim is to publish these core components as suggestions for international methodological reporting standards to make gbPM internationally comparable.
In a second step, these standards can be developed towards a methodological assessment instrument.

---

[23] Murphy MK, Black NA, Lamping DL, McKee CM, Sanderson CF, Askham J, Marteau T. Consensus development methods, and their use in clinical guideline development.Health Technol Assess. 1998;2 (3):i-iv, 1-88. Review.

[24] Jones J, Hunter D: Qualitative research: consensus methods for medical and health services research. BMJ 1995, 311:376–380.

5. Appendix

5.1 Form for 1st DELPHI-Round

Criterion Nr. /Title	
Content:	
Rationale:	
Actors (who is responsible/should participate)?	
Vote	
I agree	
I agree with modifications	
I don't agree	
Modifications/Rationale for disagreement:<br><br>Content:<br><br>Rationale:	
