# Supplementary material for: Reporting standards for guideline-based performance measures
Source: Implement Sci. 2016 Jan 15;11:6. doi: 10.1186/s13012-015-0369-z (PMC4714427; doi:10.1186/s13012-015-0369-z)
Supplement: Supplementary file 3 — G-I-N survey round I, copy of survey monkeys results and comments. (PDF 366 kb) [file 13012_2015_369_MOESM3_ESM.pdf]

3. First Criterion/Attribute 1a. The guidelines used for guideline based performance measure development are current and meet the reporting criteria published by the Guidelines International Network (G-I-N). They are selected using a validated guideline assessment tool (preferably AGREE II). 1b. The sources used for guideline based performance measure development are explicitly stated.

**answered question**	**23**
**skipped question**	**6**

I agree	I rather agree	I rather disagree	I disagree	I agree with modifications	Rating Average	Rating Count
**87.0% (20)**	8.7% (2)	0.0% (0)	0.0% (0)	4.3% (1)	3.74	23

specify what the sources were for, ie. The sources of evidence, or The sources used for evidence with which to develop guideline based performance measures were explicitly stated

8/3/2013 2:36 PMView Responses

Should be evidence-based, this should be Cleary noted in 1b, even if noted in the longer explanations under 4). By the way the notation 3.1.a, 3.1.b is confusing

7/24/2013 8:02 AMView Responses

1a) if you develop PM simulatneously to a guideline, you won't assess your own guideline with AGREE.

7/16/2013 4:25 PMView Responses

I do not think we need both the GIN criteria and AGREE II evaluation. My preference would be to stick with the established and widely accepted AGREE II tool which has tools for use available on its web site.

7/16/2013 11:48 AMView Responses

KOMMENTARE zur Rationale:

It shoudl be sought to take a more international pespective when talking citing pre-existing performance measures

8/15/2013 10:53 AMView Responses

As we ared talking about guideline based performance measure, I think that option B "additional sources" dont have place in this discussion

8/8/2013 7:30 PM View Responses

Why pick up one source for PMs (NQMCH)?

8/5/2013 11:20 AM View Responses

If this is meant to be a criterion it is quite lengthy and complex. Perhaps the language could be simplified. At the same time, it essentially repeats what was stated above in #3 so is this necessary? Or is this meant to simply provide more rationale for #3?

8/3/2013 2:36 PM View Responses

AGREE II has some limitations - for example, the applicability criteria are not helpful in distinguishing among guidelines as almost no guidelines (apart from NICE) meet these criteria - and it is time consuming and resource intensive - however, we don't have anything better. The wording of recommendations is crucial for performance measure development, as many recommendations turn out not to be measurable because they are too imprecise - point being, some criteria are more important than others. However, none of this changes my fundamental agreement with these criteria.

7/24/2013 10:33 AM View Responses

4a) any help for deciding what is up to date?

7/16/2013 4:25 PM View Responses

See previous comment about duplication in use of GIN and AGREE criteria. I find this rationalisation a bit hard to follow and repetitive. eg in 4(a) the second paragraph adds little or nothing to the items covered in the tools mentioned in the previous paragraph.

7/16/2013 11:48 AM View Responses

A suggestion to consider the possibility of selecting a high quality "adapted" CPG if it meets the mentioned criteria (with full description of the methods used for the adaptation process e.g. ADAPTE v 1.0 or 2.0 Process)

Second Criterion/Attribute: 2. Guideline recommendations selected for performance measures intended to serve for quality judgement are strong recommendations.

**answered question**	**22**
**skipped question**	**7**

Second Criterion/Attribute: 2. Guideline recommendations selected for performance measures intended to serve for quality judgement are strong recommendations.						
**I agree**	**I rather agree**	**I rather disagree**	**I disagree**	**I agree with modifications**	**Rating Average**	**Rating Count**
**45.5% (10)**	31.8% (7)	0.0% (0)	9.1% (2)	13.6% (3)	2.86	22
					Suggested modifications Show Responses	8

Modifications:

In some grading systems, if there is weak evidence but strong consensus or impact on public health, and evidence and recommendations are not separated, one might be compelled to make a weak recommendation by nature of the scoring system. These areas might still be important to measure

8/9/2013 11:47 PMView Responses

Not all use GRADE and therefore this is not an applicable criterion.

8/5/2013 12:19 AMView Responses

Since criteria differ across scoring systems perhaps be more specific with respect to what is meant by "strong". Probably not necessary to say "to serve for quality judgement" because that is what performance measures are used for.

8/3/2013 2:38 PMView Responses

strong recommendations OR .....as described below, the recommendations with an incomplete evidence base (and likely no more evidence) to see what happens.

7/24/2013 3:56 PMView Responses

Could the wording be more clear, eg "Strong recommendations (as opposed to weak recommendations) form the basis of performance measures used to judge the quality of a service."

7/24/2013 10:37 AMView Responses

The meaning of "strong" is uncertain without prior definition Strong and weak should be more clearly defined and presented in the text below

7/24/2013 8:10 AMView Responses

Many guidelines have more than just Strong and Weak recommendations. Moderate recommendations could also be selected for performance measures.

7/15/2013 5:43 PMView Responses

...are strong recommendations and/or based on high quality evidence.

7/15/2013 4:17 PMView Responses

Comments to Rationale

Because GRADE is not used at every high quality guideline organisations it could also be recommended the the relevance of the performance measurement should be tested during the development process.

8/5/2013 12:19 AMView Responses

The rationales are fine but rather wordy. I am not sure if you wanted feedback on the concepts they reflect or also the writing style. I think a wee bit more work on tightening the language, descriptions, etc. would be an asset.

7/24/2013 3:56 PMView Responses

Also, the use of strong recommendations increases the acceptability of the performance measures to the service/clinicians, particularly when they form the basis of quality assessments or targets.

7/24/2013 10:37 AMView Responses

Depending on the goals and use of the indicators not only strong recommendations might be useful, e.g. for internal quality improvement with the goal of starting discussions on internal standards.

7/24/2013 8:42 AMView Responses

The text above should be clarified and better presented with clearly identifiable key points

7/24/2013 8:10 AMView Responses

I would like to add that generally, strong recommendations can be positive or negative. The latter would result in a PM with a low reference range in order to eliminate e.g. harmfull / useless interventions.

7/16/2013 4:30 PMView Responses

Quality Indicators need to be a valid measure of quality, ie measure what it is intended that they measure. QIs which appear rational may not accurately reflect quality per se - eg increasing ascertainment will push an indicator up when desired travel is downwards, regard;ess of the strength of evidence for the intervention

7/16/2013 1:42 PMView Responses

There are both pragmatic and methodological reasons for not restricting performance measures to strong recommendations. Pragmatically, strong recommendations are comparatively rare and limiting to them will narrow the scope for performance measures relating to any given topic. Weak recommendations can and do relate to clinical questions for which there may never be strong evidence because of the unherent difficulty of conducting RCTs. Palliative care is an example of an area where this is true. Clarity about the strength of underlying recommendations is more important.

7/16/2013 11:48 AMView Responses

"This does not preclude the use of weak recommendations if the resulting performance measures are not used for judgdment but for monitoring the degree of adherence (exploring its reasons) or for internal quality management (e.g., local clinical audit). " This sentence sounds contradictory to the first two sentences and is confusing.

7/15/2013 3:34 PMView Responses

Third Criterion/Attribute 3. There is a clear and detailed description of the consensus methods used to select guideline based performance measures.

**answered question**	**22**
**skipped question**	**7**

I agree	I rather agree	I rather disagree	I disagree	I agree with modifications	Rating Average	Rating Count
**81.8% (18)**	13.6% (3)	0.0% (0)	0.0% (0)	4.5% (1)	3.68	22
				Suggested modifications Show Responses		3

Modifications

Not everyone would refer to the methods as consensus since that implies discussion and agreement and both Delphi and RAND use systematic rating approaches in addition to discussion. So perhaps: Methods used to develop or select guideline based performance measures are clearly described

8/3/2013 2:41 PMView Responses

So, it is considered that the only way to select a guideline performance measure is a consensus method? Rather limitative. Also why use the plural of measures?

7/24/2013 8:20 AMView Responses

Add also options of informal consensus in case the CPG development/adaptation group had limited resources and time

7/15/2013 10:36 PMView Responses

Comments:

I've been in some of those groups. The problem is that consensus can overwhelm evidence. I am assuming that this paragraph applies to measure development only? There should be a defined process for such development that includes criteria and templates, going beyond discussion and agreement without methods or criteria.
8/9/2013 11:48 PMView Responses

1.- The core component of RAND method is not only a "written appraisal" but a "critical appraisal" of potencial performance, with the voting process to DETEC agreement within the consensus group, whem there is not agreement, they look for another preformance measures which get aggrement between the panel. 2.- To me it is more important than the "precisión" to find the performance measure, that the selection process should be explicitly described.
8/8/2013 7:43 PMView Responses

I don't quite understand the rationale, this seems moore like the descrption of present state of consensus methods
8/5/2013 12:22 AMView Responses

I think we need to see more examples of this (detailed description of consensus methods) published, and perhaps we need detailed reporting standards for this aspect, as it is not necessarily done to a consistent standard internationally.
7/24/2013 10:39 AMView Responses

The criteria used for the consensus method are crucial. Several modifications of the RAND/UCLA method use unspecific or overburdened criteria which cannot be distintctive an lead to invalid results, e.g. exclusion of primary end points as not relevant. Standardized criteria should be developed.
7/24/2013 8:43 AMView Responses

Some consider that the RAND/UCLA is not a consensus method.
7/24/2013 8:20 AMView Responses

is the third criterion focussing on consensus finding? or on assessing potenial PM? or both?
7/16/2013 4:38 PMView Responses

Delphi method can give rise to 'seems reasonable' type judgements rather than critically reviewing the validity and robustness of an indicator
7/16/2013 1:44 PMView Responses

Fourth Criterion/Attribute 4. Developers demonstrate that they have considered the following attributes within the development process of guidelines based performance measures before using them: • Relevance (at least: content validity) • Scientific Soundness (at least: the evidence supporting the measure is explicitly stated) • Feasibility (at least: clarity of definition)

**answered question**	**21**

Fourth Criterion/Attribute 4. Developers demonstrate that they have considered the following attributes within the development process of guidelines based performance measures before using them: • Relevance (at least: content validity) • Scientific Soundness (at least: the evidence supporting the measure is explicitly stated) • Feasibility (at least: clarity of definition)						
					skipped question	8
I agree	I rather agree	I rather disagree	I disagree	I agree with modifications	Rating Average	Rating Count
**61.9% (13)**	19.0% (4)	4.8% (1)	0.0% (0)	14.3% (3)	3.14	21
					Suggested modifications Show Responses	6

Modifications

Beyond stating the evidence, the guideline should demonstrate how the evidence was applied and what the rationale for the recommendation was. We need to be able to determine logic and common sense used, not just a knowledge dump. Also need to be sure that the evidence has been critically appraised properly. This is still a murky area in many cases
8/9/2013 11:52 PMView Responses

Relevance: content validity in terms of patient outcomes
8/8/2013 7:45 PMView Responses

To me relevace and content validity are different things. Both relevance and validity should be considered! Explicit description for these words are needed before agreeing or disagreeing. See also my earlier comment on strong recommendations
8/5/2013 12:27 AMView Responses

Suggest calling it Clincal or Health System relevance. Scientific soundness. I think you should define all the bits of scientific soundness you think are relevant.
7/24/2013 4:02 PMView Responses

Scientific soundness reads strange!
7/24/2013 8:24 AMView Responses

...evidence supporting the measure *as a valid measure of quality* is explicitly stated. Need to be explicit whether actually measuring 'quality' or failures ('lack of quality') - former is more challenging, like 'measuring safety' as opposed to measuring patient harm - see recent Health Foundation publication
7/16/2013 2:54 PMView Responses

Comments:

See above - this generally means going beyond structure and looking at how the process was applied
8/9/2013 11:52 PMView Responses
- reliability - validity - which types - I think going beyond content or face validity might be important - sensitive to change
7/24/2013 4:02 PMView Responses
I am not sure about the statement that technical feasibility "should not primarily be used as 'knock out' criterion" - presumably this means as an exclusion criterion. I am not sure why this is being stated. Surely in most cases non-feasibility of data collection would be an appropriate exclusion criterion - what is the point of specifying a performance measure for which data can almost certainly not be collected?
7/24/2013 10:43 AMView Responses
Relevance is often used as a "general criterion" which in fact only states face validity (asseses "good indicator" vs "bad indicator".) Relevance should only be used as "relevance" (does the indicator adress an important aspect of care). An important criterion is "can the result be influenced by the caregiver?" This seems to be a first line criterion. Scientific soundness and feasibility are unspecific criteria too which can be misleading. In my opinion it is better to use more specific criteria in the first line. A second assessment can be done when the indicator is in pracitacal use.
7/24/2013 8:48 AMView Responses
Very dense text, difficult to find the key messages. In my opinion clear definitions, statements should be provided here. Improve the presentation of these long blocks of text
7/24/2013 8:24 AMView Responses
I don't agree with everything written at 10. But it doesn't make sense to discuss it in this box :-)
7/16/2013 4:44 PMView Responses
Outcome measures can monitor quality, but do not of themselves identify where the deficits are; process measures indicate where intervention is required to improve quality of care - the two are distinct and have different utilities. .
7/16/2013 2:54 PMView Responses

Fifth Criterion/Attribute 5. Denominator and Nominator of the guideline based Performance Measure are specified unambiguously and in detail.

**answered question**	**21**
**skipped question**	**8**

Fifth Criterion/Attribute 5. Denominator and Nominator of the guideline based Performance Measure are specified unambiguously and in detail.

I agree	I rather agree	I rather disagree	I disagree	I agree with modifications	Rating Average	Rating Count
**81.0% (17)**	9.5% (2)	0.0% (0)	0.0% (0)	9.5% (2)	3.52	21
				Suggested modifications Show Responses		3

numerator
8/3/2013 2:43 PMView Responses
"nominator" correct term in English is "numerator" and they are usually stated in the opposite order - "numerator and denominator"
7/24/2013 10:51 AMView Responses
.
7/15/2013 5:47 PMView Responses

Agree that this is a crucial criterion.
7/24/2013 10:51 AMView Responses
Give a clear example with proper numerator and denominator
7/24/2013 8:26 AMView Responses
What about weighted or standardised metrics?
7/16/2013 2:55 PMView Responses
I find this difficult to follow. An example would be a great help.
7/16/2013 11:50 AMView Responses
This is the ideal but, in practice, is not always possible
7/15/2013 5:47 PMView Responses

Sixth Criterion/Attribute 7. Indicators intended for use for regional or national reporting purposes or pay for performance are piloted prior to their recommendation for use.

**answered question**	**21**

Sixth Criterion/Attribute 7. Indicators intended for use for regional or national reporting purposes or pay for performance are piloted prior to their recommendation for use.

					**skipped question**		**8**
**I agree**	**I rather agree**	**I rather disagree**	**I disagree**	**I agree with modifications**	**Rating Average**	**Rating Count**
**47.6% (10)**	33.3% (7)	4.8% (1)	4.8% (1)	9.5% (2)	3.05	21
					Suggested modifications Show Responses	6

Especially if used nationally or for pay for performance

8/5/2013 12:35 AMView Responses

Ideally this would be great. The challenge is the cost of creating these performance measures if using clinical data base systems may not permit a pilot. You either do it or not do it. Its not ideal but I am not convinced this is a realistic criterion.

7/24/2013 4:04 PMView Responses

it is not necessary to pilot an indicator in a measurement field with several indicators in use where only one more indicator is added.

7/24/2013 8:50 AMView Responses

Sentence should be shortened to "indicators should be piloted...

7/24/2013 8:27 AMView Responses

piloted or subject to rapid cycle small test of change (PDSA) quality improvement approach

7/16/2013 2:57 PMView Responses

Even if not already piloted; a tool published for national/international evaluation can be used (which then would be utilization and active participation in the actual piloting)

7/15/2013 11:22 PMView Responses

Comments:

Unintended consequences can take several years to become apparent - longer than most pilot periods. I agree piloting is ideal, but it won't happen in many situations because of time and resource constraints.

Seventh Criterion/Attribute 7. There is a clear description of the intended use of the performance measures (quality improvement, quality assurance with or without accountability purposes, pay for performance) and at what level (local, regional, national).

**answered question**	**21**
**skipped question**	**8**

I agree	I rather agree	I rather disagree	I disagree	I agree with modifications	Rating Average	Rating Count
**76.2% (16)**	23.8% (5)	0.0% (0)	0.0% (0)	0.0% (0)	3.76	21
				Suggested modifications		0

Comments:

A well designed performance measure is independent of the use you could give to it.
8/8/2013 7:49 PMView Responses

Intended setting should also be noted e.g. primary care, hospitals/secondary care etc.
8/5/2013 12:38 AMView Responses

those developing performance measures may have one use while others may apply them or use them differently so this criterion is of less importance than others
8/3/2013 2:46 PMView Responses

This is a helpful distinction to make, particularly because it affects the reception of the measures by the service/clinicians
7/24/2013 10:51 AMView Responses

maybe also add declaration of the orderer/ prinicipal of the PM? (if not included ina separate criterion)
7/15/2013 3:39 PMView Responses

Performance Measures in use are regularly reviewed. Criteria for deciding to change or stop using a specific performance measure are stated.

**answered question**	**21**
**skipped question**	**8**

I agree	I rather agree	I rather disagree	I disagree	I agree with modifications	Rating Average	Rating Count
**85.7% (18)**	4.8% (1)	4.8% (1)	0.0% (0)	4.8% (1)	3.67	21
				Suggested modifications Show Responses		2

Comment:

There are almost no publications on the issue of retirement of indicators. More discussion here is needed. However, it should be acknowledged that the system of retirment is closely linked to the system of reviewing and developing indicators. There might be remarkable differences between countries.
8/15/2013 10:53 AMView Responses

while this makes sense on a practical level, it is unclear how this would be operationalized and by who
8/3/2013 2:47 PMView Responses

May need to take account of need to improve over time - not just a simple static target
7/16/2013 2:59 PMView Responses

The composition of the panel deciding on guideline based performance measures includes relevant stakeholders in the field, experts in quality measurement and patient representatives.

The composition of the panel deciding on guideline based performance measures includes relevant stakeholders in the field, experts in quality measurement and patient representatives.

**answered question**	**21**
**skipped question**	**8**

I agree	I rather agree	I rather disagree	I disagree	I agree with modifications	Rating Average	Rating Count
**61.9% (13)**	14.3% (3)	0.0% (0)	0.0% (0)	23.8% (5)	2.90	21
				Suggested modifications Show Responses		6

In some subjects such as mental health also patient-familiy members should be involved.

8/15/2013 10:53 AMView Responses

I am not sure if patient representatives are needed.

8/5/2013 12:38 AMView Responses

"patient representatives who have been appropriately trained and supported to participate meaningfully in the panel"

7/24/2013 10:51 AMView Responses

Also include specifically the clinicians involved

7/24/2013 8:29 AMView Responses

Patient representatives should always provide input but do not necessarily need to be part of each and every panel that makes the decisions. Panel should be multidisciplinary.

7/15/2013 5:55 PMView Responses

I would not necesserely include patient representatives in the panel. However, the aspects important for the patients should be taken into consideration for example by consulting their representatives.

7/15/2013 4:30 PMView Responses

Comments:

I think it is important to include data management experts in a group to help ensure

data can be collected effectively in the health care settings in which the measures are to be used.

7/16/2013 11:53 AM View Responses

Is there evidence that having patients be full participants in the panel always improves the measures that are developed?

7/15/2013 5:55 PM View Responses

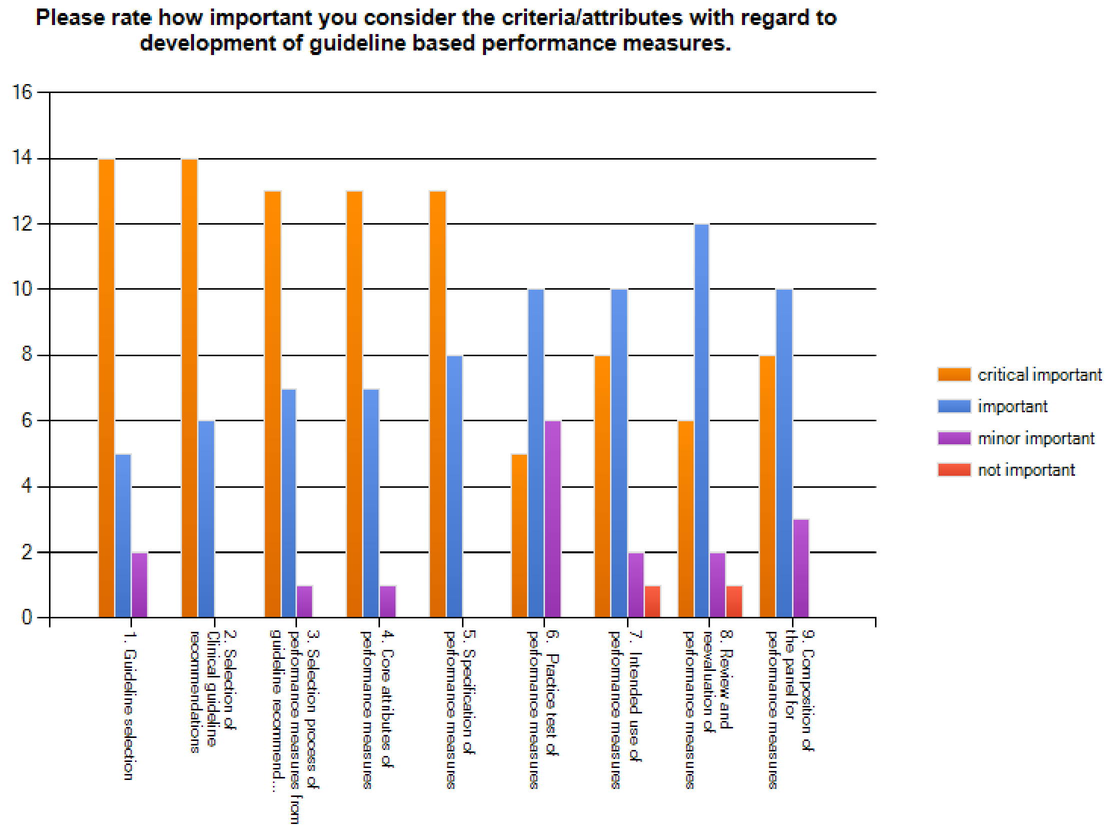

Please rate how important you consider the criteria/attributes with regard to development of guideline based performance measures.

**answered question**	**21**
**skipped question**	**8**

Please rate how important you consider the criteria/attributes with regard to development of guideline based performance measures.

	critical important	important	minor important	not important	Rating Count
**1. Guideline selection**	**66.7% (14)**	23.8% (5)	9.5% (2)	0.0% (0)	21
**2. Selection of Clinical guideline recommendations**	**70.0% (14)**	30.0% (6)	0.0% (0)	0.0% (0)	20
**3. Selection process of performance measures from guideline recommendations**	**61.9% (13)**	33.3% (7)	4.8% (1)	0.0% (0)	21
**4. Core attributes of performance measures**	**61.9% (13)**	33.3% (7)	4.8% (1)	0.0% (0)	21
**5. Specification of performance measures**	**61.9% (13)**	38.1% (8)	0.0% (0)	0.0% (0)	21
**6. Practice test of performance measures**	23.8% (5)	**47.6% (10)**	28.6% (6)	0.0% (0)	21
**7. Intended use of performance measures**	38.1% (8)	**47.6% (10)**	9.5% (2)	4.8% (1)	21
**8. Review and reevaluation of performance measures**	28.6% (6)	**57.1% (12)**	9.5% (2)	4.8% (1)	21
**9. Composition of the panel for performance measures**	38.1% (8)	**47.6% (10)**	14.3% (3)	0.0% (0)	21

**Please rate how important you consider the criteria/attributes with regard to development of guideline based performance measures.**	
Suggested additional criteria/attributes you consider essential	0
