# Supplementary material for: Reporting standards for guideline-based performance measures
Source: Implement Sci. 2016 Jan 15;11:6. doi: 10.1186/s13012-015-0369-z (PMC4714427; doi:10.1186/s13012-015-0369-z)
Supplement: Supplementary file 4 — G-I-N survey round 2; copy of results. (PDF 266 kb) [file 13012_2015_369_MOESM4_ESM.pdf]

GIN Reporting Standards: Final Version 12_13 (Second DELPHI round)
Invited: 27; participated: 12

1. **Criterion/Attribute**

a. State the currency of guidelines used for guideline based performance measure development and state if the guideline meets the criteria set out by the Guidelines International Network (G-I-N). Describe the guideline quality using a validated guideline assessment tool, such as AGREE II. b. Indicate additional sources, if used and the rationale for their use.

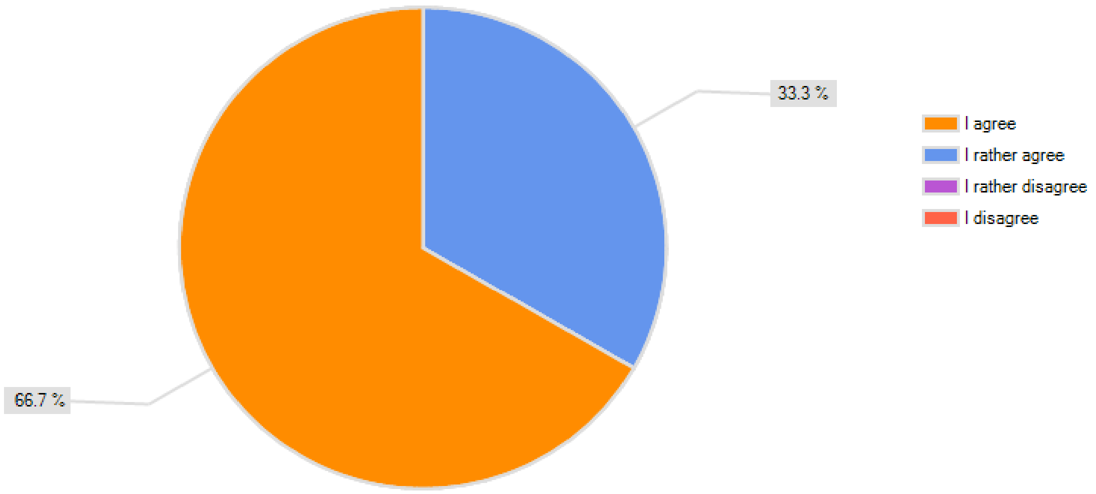

2. Criterion/Attribute

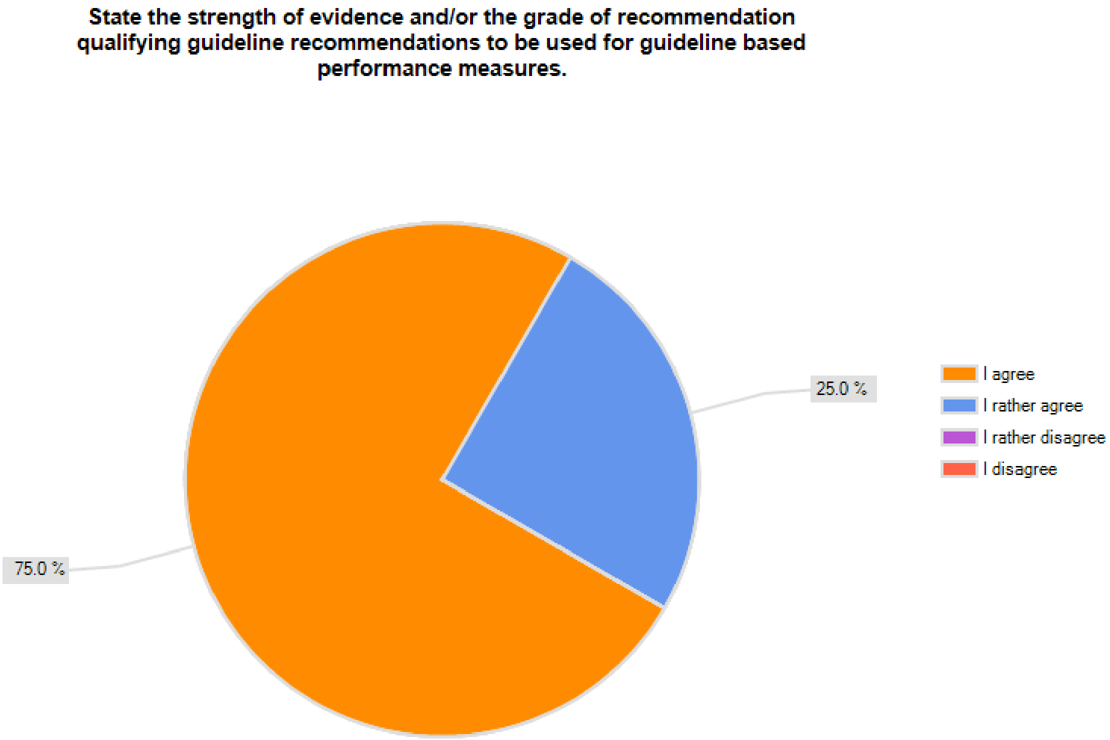

3. Criterion/Attribute:

Describe clearly and in detail the methods used to develop the performance measures from the supporting clinical guideline recommendations.

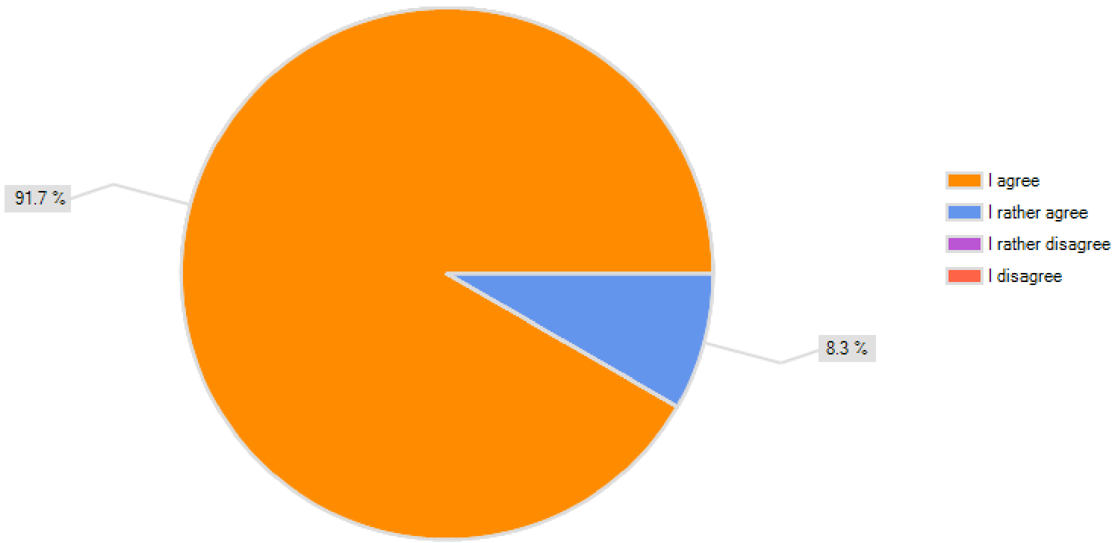

4. Criterion/attribute:

State, if the following attributes within the development process of guidelines based performance measures were considered: • Relevance (as a minimum: potential for improvement/clinical relevance)• Scientific Soundness (as a minimum: the evidence supporting the measure)• Feasibility (as a minimum: clarity of definition and measurability)

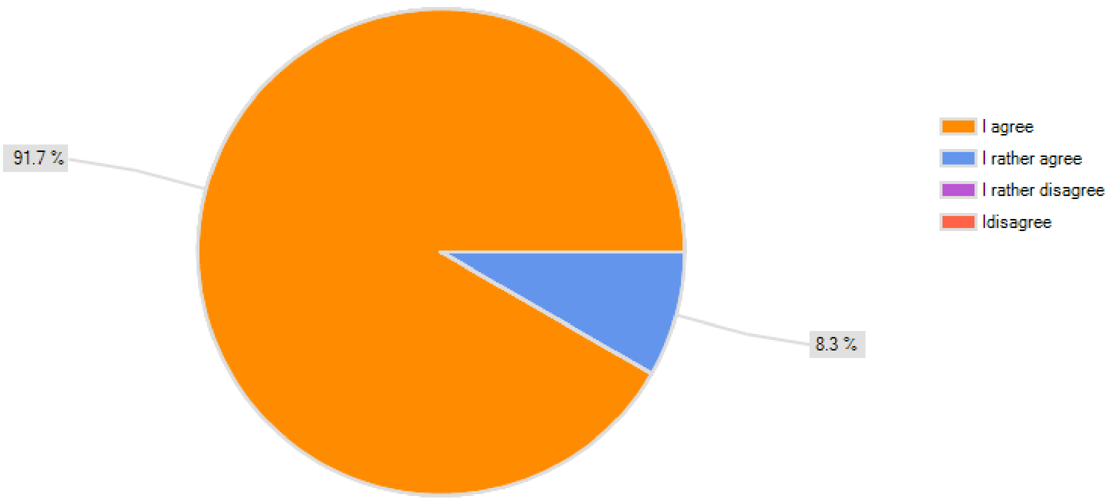

5. Criterion/Attribute

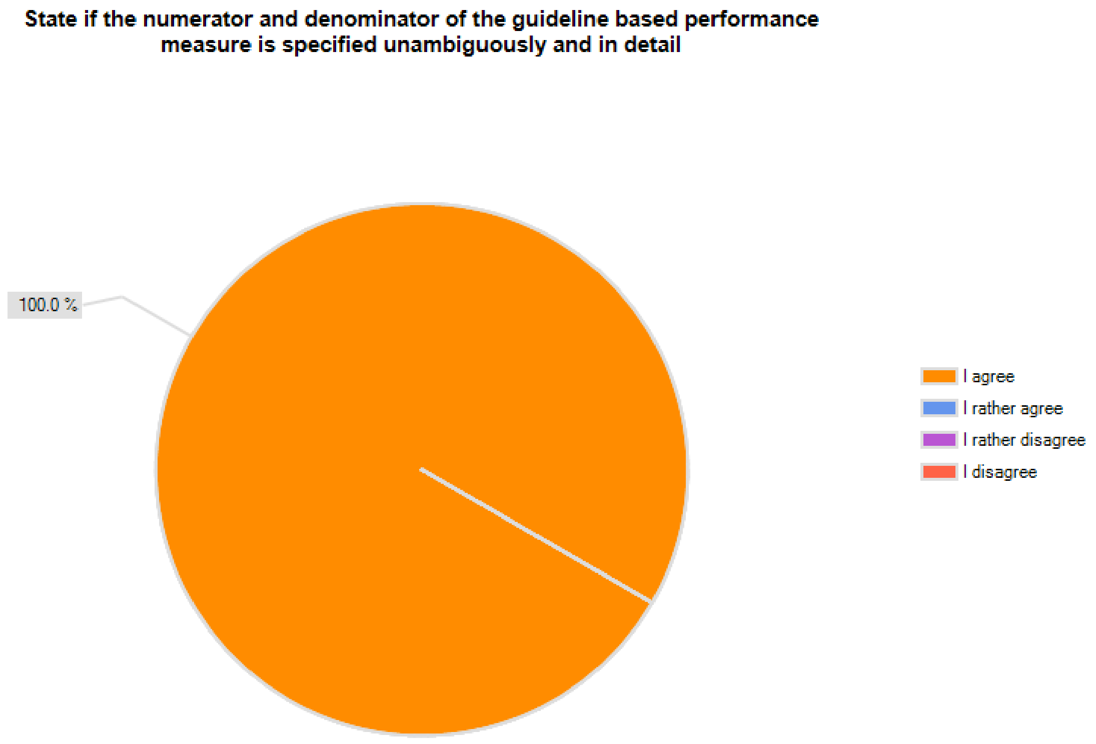

State if there is a clear description of the intended use of the performance measure (quality improvement, quality assurance with or without accountability purposes, pay for performance) and at what level in the health system it is used (local, regional, national).

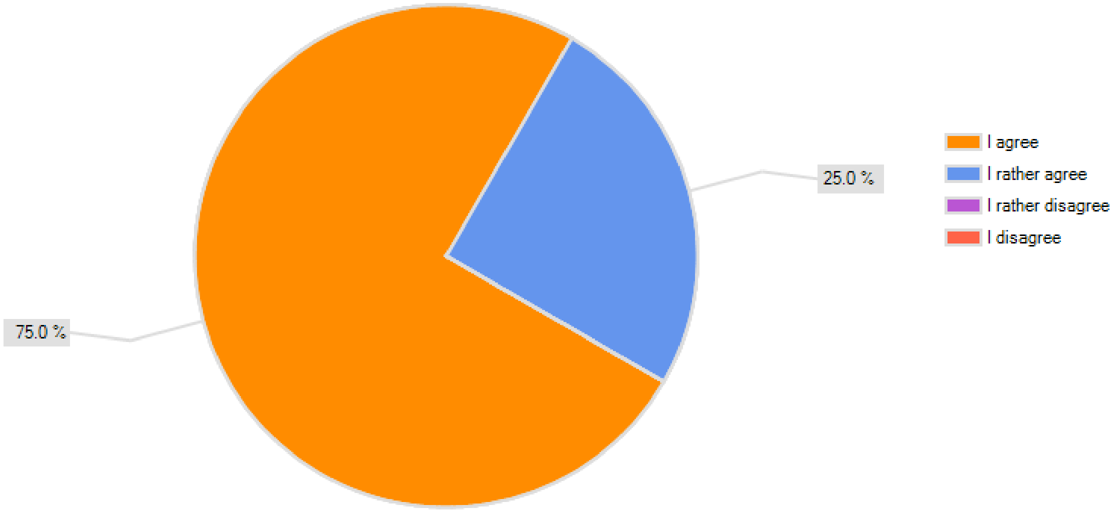

Criterion/Attribute

6. **Criterion/Attribute**

7. Criterion/Attribute

If a practice test ("piloting") is carried out prior using the guideline based performance measure, provide a full description of the process. If no practice test is done provide the reason therefore. Provide information about any other validation process in use.

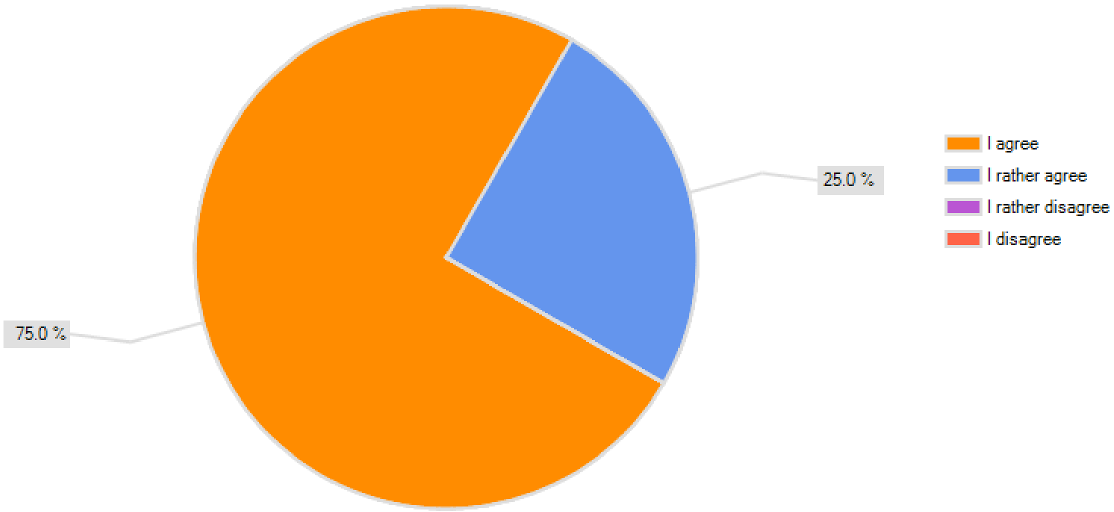

8. **Criterion/Attribute**

Report the currency of the performance measures in use. State the criteria for deciding to change or stop using performance measures.

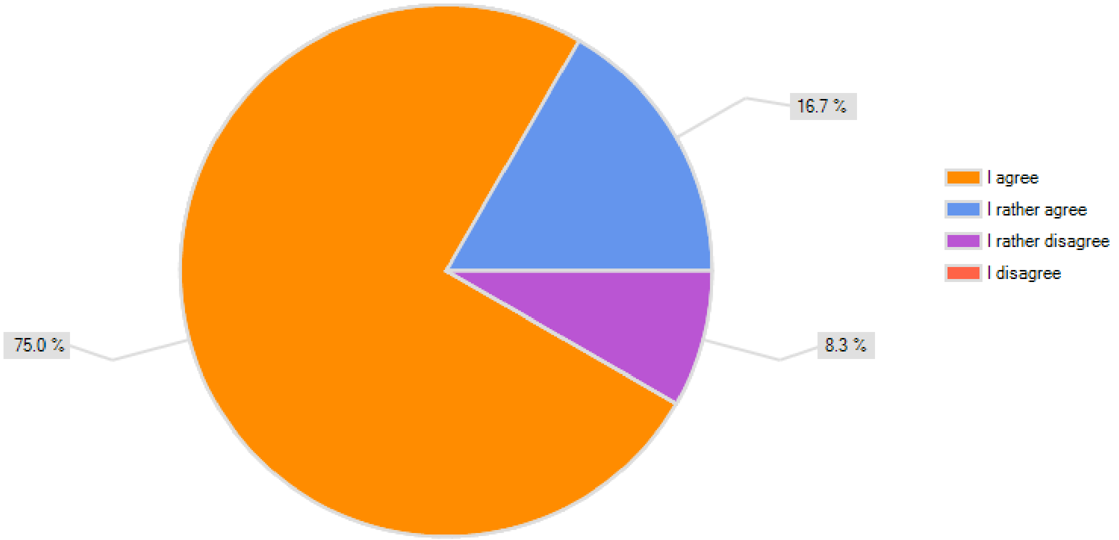

9. Criterion/Attribute

Describe clearly the composition of the panel deciding on guideline based performance measures with information on participation of multidisciplinary experts, stakeholders in the field, experts in quality measurement and patient representatives.

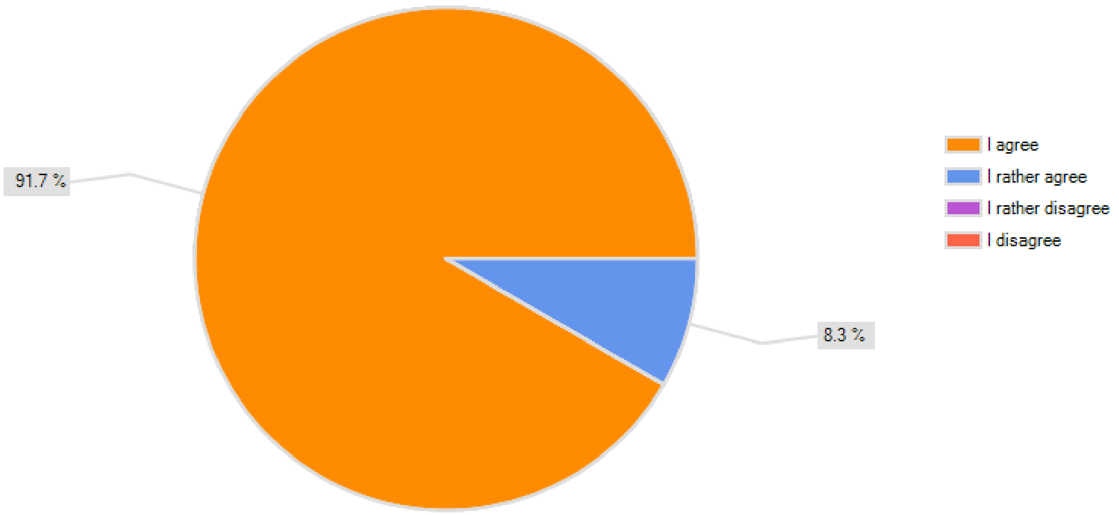

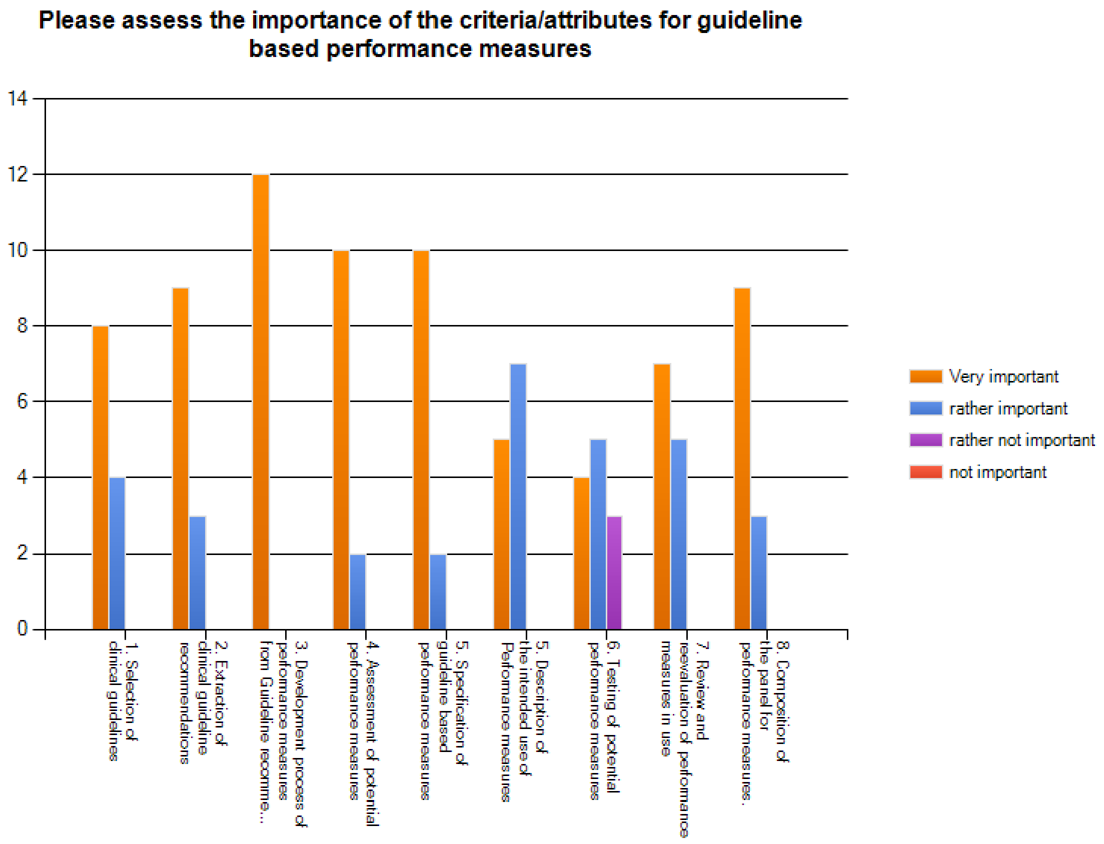

Please assess the importance of the criteria/attributes for guideline based performance measures
0
2
4
6
8
10
12
14
1. Selection of clinical guidelines
2. Extraction of clinical guideline recommendations
3. Development process of performance measures from Guideline recomme...
4. Assessment of potential performance measures
5. Specification of guideline based performance measures
5. Description of the intended use of Performance measures
6. Testing of potential performance measures
7. Review and reevaluation of performance measures in use
8. Composition of the panel for performance measures.
Very important
rather important
rather not important
not important
